# Detection of Cystatin C biomarker for clinical measurement of renal disease by developed ELISA diagnostic kits

**DOI:** 10.1186/1479-5876-12-205

**Published:** 2014-09-12

**Authors:** Renren Jiang, Chao Xu, Xiaoli Zhou, Tianhao Wang, Gang Yao

**Affiliations:** Key Laboratory for Food Safety Research, Institute for Nutritional Sciences, Shanghai Institutes for Biological Sciences, Chinese Academy of Sciences, Shanghai, 200031 China; Shanghai Normal University College of Life & Environmental Science, Shanghai, 200234 China; Key Laboratory for Food Safety Risk Assessment, Ministry of Health, Beijing, 100021 China; School of Perfume and Aroma Technology, Shanghai Institute of Technology, Shanghai, 201418 China; Department of General Practice, Zhongshan Hospital, Fudan University, Shanghai, 200433 China

**Keywords:** Kidney, Human cystatin C, Renal function, VHH, ELISA

## Abstract

**Background:**

Human cystatin C (HCC) is a potential biomarker for tubular damage and impaired renal function. It is difficult to obtain efficient paired monoclonal antibodies against HCC with low molecular to meet the requirements for clinical application The present study was to establish a stable and repeatable measurement for HCC with self-made monoclonal antibodies (McAbs) and Variable domain of heavy chain of heavy-chain antibody (VHHs) increase the sensitivity.

**Methods:**

With hybridoma technology and phage display technology: R-HCC as a screening antigen and N-HCC as the detector for antigens to obtain the specific antibody and established an enzyme-linked immunosorbent assay for human cystatin C using self-made McAbs and VHHs.

**Results:**

We have successfully obtained three McAbs; 5 F2, 4E4, 1E11 and four VHHs; 3-2, 3-24, 3-33 and 4-5 which were specific for HCC. The measurement of HCC was established with the self-made monoclonal antibodies and VHHs with a high sensitivity the lower limit of detection at 0.5 ng/ml and the detection range at 0.5 ~ 31.3 ng/ml.

**Conclusion:**

Our data provides a new approach for paired antibody screening and testing of the small molecular biomarker with a single dominant epitope, with the important biological and clinical significance.

## Background

Renal insufficiency is an important influencing factor for the prognosis of patients with chronic heart failure and more accurate detection of mild renal impairment may improve the risk stratification of the patients, especially with the early impairment of renal function. Circulating levels of creatinine are considered as one of the common readouts to estimate glomerular filtration rate (GFR), an important evaluation index of renal function [1–4]. Circulating levels and endogenous clearance of creatinine are used to clinically detect GFR, while there are many factors influencing the accuracy [5, 6]. Some reports of early nephropathy demonstrated that cystain C has high sensitivity and specificity in glomerular filtration rate detection [7, 8]. Cystatin C, a non-glycosylated protein, is produced continuously by all cells in organs/tissues. It is filtered in the renal glomeruli and completely reabsorbed by the renal tubuli. Alterations of serum cystatin C were considered as an early renal marker in diabetic patients [8–11], cardiovascular diseases kidney transplantation, hyperthyroidism, cancer, or others [12–15]. The detection of cystatin C was further improved for early diagnosis of serious diseases, with the potential of social and economic significance [16–20].

Cystatin C as the primary biomarker to estimate GFR and kidney function was measured in serum, plasma, cerebrospinal fluid, or urine [21–26]. The aim of the present study was to establish a new Double-Antibody-Sandwich Enzyme-Linked immunosorbent assay(DAS-ELISA)-based measurement of HCC with the self-made monoclonal antibody and VHHs by applying the hybridoma technology and phage VHH display technology, to develop a HCC ELISA Test Kit with the highest sensitivity, low cost, and easy operation.

## Methods

### Reagents and instruments

Nucleic acid gel imaging system, nucleic acid electrophoresis apparatus and protein gel electrophoresis apparatus were purchased from Shanghai Tanon company. Microplate reader was purchased from Thermo Fisher Technology Ltd (Shanghai, China). Polyethylene glycol(PEG) was purchased from Merck Co, Mouse typerisotyping panel kit from Bio-RAD Co, and RPMI MEDIEM 1640 medium, penicillin-Streptomycin double antibody solution, newborn calf serum and HEPES from Life Technologies Gibco Co. Hypoxanthine-Aminopterin-Thymidine (HAT) supplemented medium, Hypoxanthine- Thymidine (HT) supplemented medium, Freund's complete or incomplete adjutants were purchased from Sigma. Natural human cystatin C (N-HCC) was purchased from Enzo Life Sciences Ltd., Horseradish peroxidase-conjugated goat anti-mouse IgG from Santa Cruz Biotechnology Inc., or Tween-20 and Bovine serum albumin (BSA) from Amresco. BALB/c mice were from Shanghai Institutes for Biological Nutrition, according to the ethical permission approved by the committee of Animal Ethical Evaluation, Chinese Academy of Science. The natural camel single-domain heavy chain antibody library was kindly provided by Dr. Ario de Marco for Italian IFOM-IEO center.

### Preparation of recombinant HCC

The total RNA was extracted from renal epithelial 293 T cells using the TransZol Up RNA kit. The cDNA was synthesized from RNA using the Superscript II reverse transcriptase with OligodT (18) primers, as the template for the PCR reaction. The primers specific for HCC were used to introduce the restriction sites BamH I and Xho I (The primers: 5’-GGATCCAGTCCCGGCAAGCCG-3’ and 5’-CCTCGAGCTAGGCGTCCTGACAGGT-3’). PCR products (363 bp) corresponding to HCC fragments and then connected to pEASY-T1 simple T vectors [27–29]. The cloning T vectors which contain purpose gene and the prokaryotic expression vector pET-32a was digested with BamH I and Xho I twice and dephosphorylated and gel purified before the ligation incubation. The ligation was performed overnight at 16°C by T4 DNA ligase. The recombinant plasmids were transformed into Rosetta and the transformants were selected on Luria-Bertani LB agar plates supplemented with 100 μg/ml ampicillin. Single bacterial colony was picked from the transformned plate and verified by PCR, and the positive bacteria were induced to express the target protein. The positive single colonies inoculated (1:100) into 10 ml of LB liquid media containing 100 μg/ml ampicillin as appropriate. Bacterial cultures were incubated at 37°C overnight with shaking and then inoculated into 1 L of fresh antibiotic-containing Luria-Bertani (LB). Isopropyl thio-β-D-galactose glycoside (IPTG) was added to a final concentration of 0.5 mM to induce protein expression. Bacteria were harvested by centrifugation (8000 r/min, 10 min, 4°C) and pellets were re-suspended in 100 ml pre-cold PBS, where phenylmethanesulfonyl fluoride(PMSF) was added at 1 mM. Bacterial lysate were centrifuged (12,000 r/min, 15 min, 4°C) and the supernatants were transferred to new tubes, while the precipitates were re-suspended in PBS [27, 30–33]. An appropriate amount of both the supernatants and suspensions were mixed with an equal volume of 2 × SDS-PAGE (sodium dodecyl sulfate polyacrylamide gel electrophoresis) loading buffer. Expression levels of recombinant R-HCC in supernatants and precipitates were analyzed by SDS-PAGE analyses. Bacterial samples that were not induced with IPTG, wild-type Rosetta transformed with pET-32a were used as controls.

The proteins were expressed in an insoluble form as inclusion bodies, where 2 M urea was added, ultrasounded, and hen the lysates were centrifuged (12,000 r/min, 20 min, 4°C). The supernatants were transferred to dialysis bags in PBS and dialyzed overnight at 4°C thrice. The protein solution was dialyzed at the renaturation of His-R-HCC more than 80% (wt/wt). The Ni-NTA agarose (Qiagen) was used to purify His-R-HCC according to the manufacturer’s instructions. Two ml of Ni-NTA agarose pre-equilibrated with the lysis buffer, including 50 mM NaH_2_PO_4_, 300 mM NaCl, 10 mM imidazole, PH 8.0, was added to the supernatant and gently mixed at 4°C for 1 h. The lysate agarose mixture was loaded to the column and washed with the buffer, including 50 mM NaH_2_PO4, 300 mM NaCl, and 0.05% (v/v) Tween 20, at pH 8.0, by the stepwise addition of graduate imidazole concentrations at 20 mM, 50 mM, 100 mM, 200 mM, or 500 mM, respectively. The protein was eluted using 10 ml of elution buffer with 50 mM NaH_2_PO_4_, 300 mM NaCl, 250 mM imidazole at pH 8.0. Concentrations of purified recombinant proteins were determined by the Bradford assay [30]. His-tagged proteins were digested by thrombin and then the ultrafiltration concentrate was analyzed by SDS-PAGE analyses and Western blot.

### Mouse immunization and evaluation of anti R-HCC sera

Four BALB/c female mice (6-8 weeks old) were immunized with R-HCC by intraperitoneal injections of 100 μL at the concentration of 1 g/L of R-HCC and 100 μL of Freund's adjuvant. The first doses contained complete Freund's adjuvant, and subsequent doses were given a 3-weeks interval using incomplete Freund's adjuvant. Blood samples were collected one week after the fourth injection, and the titer and specificity of antibody response were determined with indirect ELISA [34, 35].

### Cell fusion and hybridoma selection and cloning

Murine myeloma cells were cultured in high-glucose Dulbecco's minimal Eagle's medium (DMEM) supplemented with 20% (v/v) FBS and 1% (wt/vol) penicillin-streptomycin. Cell fusion procedures were carried out according to the protocols of the Clonal Cell-HY™ kit. Mouse spleen lymphocytes were mixed with the myeloma cells at the ratio of 5:1 using 1 ml of PEG 4000. The fused cells were then transferred into a tissue culture plate containing the methycellulose-based selection medium and incubated. The individual hybridoma clones in the semisolid medium were transferred into a 96-well plate about 12 days after the cell fusion. The supernatants were collected when hybridoma cells were grown to approximately 10-20% (v/v), and initial screening was performed using indirect ELISA. The screen yielded positive hybridoma cells, which were subsequently sub-cloned thrice by the limiting dilution. Aliquots of first hybridomas and the clones were cryopreserved at several stages during the development.

### Purification and characterization of monoclonal antibodies (McAbs)

Female BALB/c mice were intraperitoneally injected with 10^6^ hybridoma cells 7 days after intraperitoneal injection with 0.5 ml of liquid olefin. After 14 days, ascites fluid was collected 14 days after the injection with cells and centrifugated at 4000 r/min for 15 min. McAbs were purified from mouse ascites by ammonium sulfate precipitation followed by affinity chromatography on a protein G column [30, 34–37]. Classes and subclasses of McAbs were identified by mouse monoclonal antibody isotyping reagents (Sigma-Aldrich) following the manufacturer’s instructions [33].

### Determination of McAbs Affinity

The affinity constants of McAbs were detected after sample preparation, chip surface pretreatment, sample injection, regeneration, and data analysis. Three μg of natural Human cystatin C (N-HCC) and 0.05 M PBS at 7.4 was prepared and filtered. Our pilot experiments showed that the isoelectric point of 9.0 HCC was bonded to the surface of CM5 chip in HCl-glycine buffer at pH = 4.5. Three μg HCC was dissolved in 200ul of HCl-glycine buffer to be fully integrated on the CM5 sensor, and then equilibrated with PBS overnight. The chip was regenerated and then finished, followed by data analysis.

### Selection of phage displayed VHHs

R-HCC was coated overnight at 4°C in 4 ml Nunc-Immuno™ or Maxisorp™ tubes at a concentration of 30 μg/ml using 50 mM sodium carbonate buffer thrice before the addition of 3 × 10^15^ phages for the first round of panning after the blocking. Tubes were washed 10 times with PBS 0.05% Tween and PBS after 30 min rocking and 90 min standing upright at room temperature, and bound phages were eluted with 10 mM HCl, pH 2.0. Eluted phages were neutralized by Tris–HCl, pH 8.0 and then used to infect TG1 cells at 37ºC for 40 min. Infected cells were harvested by centrifugation and The new sublibrary of phages was resuspended, titrated, and used in the second round of panning [38, 39]. The procedure was repeated and the enrichment of the phage sublibrary obtained was calculated as the ratio of output/input phages.

### Screening of VHHs by ELISA

Individual colonies from the dilution series were tested for antigen binding by phage ELISA [38, 39]. Tubes containing 1 ml of 2× TY medium were inoculated with 10 μl of the overnight culture. After then, 2 × 10^12^ KM13 helper phages were added, mixed, and incubated for 1 h. Discard supernatant and resuspend pellets in 5 ml of 2 × TY medium, incubated for about 18 h, and then separated and transferred. The phage clones were tested as described previously [34, 39]. Maxisorp 96-well plates (Nunc) were coated with HCC and added the antigen at 1 μg/ml. Plates were washed with PBST and treated with anti-M13 McAb HRP conjugated for 1 h at 37°C, and added with 100 μl of TMB solution after PBST washing. Plats were analysed at 450 nm in a microplate reader. Clones with an absorbance value highest were considered positive and the sequences analyzed to identify unique binders.

### Purification and characterization of VHHs

Clones with unique sequence were subcloned into pET-28a vector to obtain His-tagged recombinant VHH binders after transformation into Rosetta competent cells. The supernatant was purified by nickel column affinity chromatography directly [30, 39]. The purity of the VHH solutions was confirmed by 12% SDS gel electrophoresis and coomassie blue staining. The property of the purified VHH was determined by ELISA and Western Blotting.

### Determination of VHHs’ Affinity

The affinity of VHHs was determined as described previously [34, 39]. Plates were coated with N-HCC after blocking and washing. The antibody solution at concentrations below dissociation constant(Kd), 0.5 nM was incubated with increasing concentrations of R-HCC from 0.1 nM to 1 μM. N-HCC-coated plates were added by 10 μl of the reaction mixtures, washed with PBST, and added with 100 μl of TMB solution for the readout at 450 nm.

### The establishment of DAS-ELISA

The optimized concentrations of HCC paired antibody with McAb 5 F2 and 1E11, or phage displayed monoclonal VHHS P-3-2, P-3-24, P-3-33, and P-4-5, were determined by sandwich ELISA. McAbs at 5 μg/ml was added and the absorbance at 450 nm was measured.

### Assay optimization

The concentration of the coating conjugates with McAbs and VHH phages was optimized by orthogonal test titration. The capture antibody coated concentrations at 10.0, 5.0, 2.5, 1.0, 0.5, 0.2, and 0.1 μg/ml were used and the incubation concentrations of HCC antigen solution were 20 ng/ml. HRP-antibody solutions were diluted to 100, 200, 500, 1000, 2000, and 4000. The one with the OD450nm value closest to 1.0 was selected as the optimal detection concentration and for further assay development.

### Determination of assay properties

The properties of the developed assay, e.g. linear range, sensitivity, accuracy and precision, were validated. The linear range and sensitivity was determined according to the making of the standard curve. The HCC standard solution was diluted into 62.5, 31.3, 15.6, 7.8, 3.9, 2.0, 1.0, 0.5, 0.2, and 0 ng/ml, respectively. The standard curve was made using the screened 5 F2-P-3-2 detection kit to detect HCC concentrations. The accuracy of the kit was assayed and calculated by adding HCC to a 5-fold diluted preparation the urine or serum at standard concentrations, and detecting the recovery of HCC. The concentration of HCC in serum samples was also measured by the turbidimetric immunoassay as the reference. The concentration of HCC in serum samples measured by the commercialized kit was 0.63 μg/ml, while the DAS-ELISA could detect the serum levels of 10, 25, 50, 100, 250, 500, and 1000 fold dilution. The precision of the DAS-ELISA was calculated with the intra-variability at 30, 20, 10, or 5 ng/ml of HCC sample solution for 10 times and the coefficient of variation(CV) was used to represent the precision.

## Results

**Figure 1:**
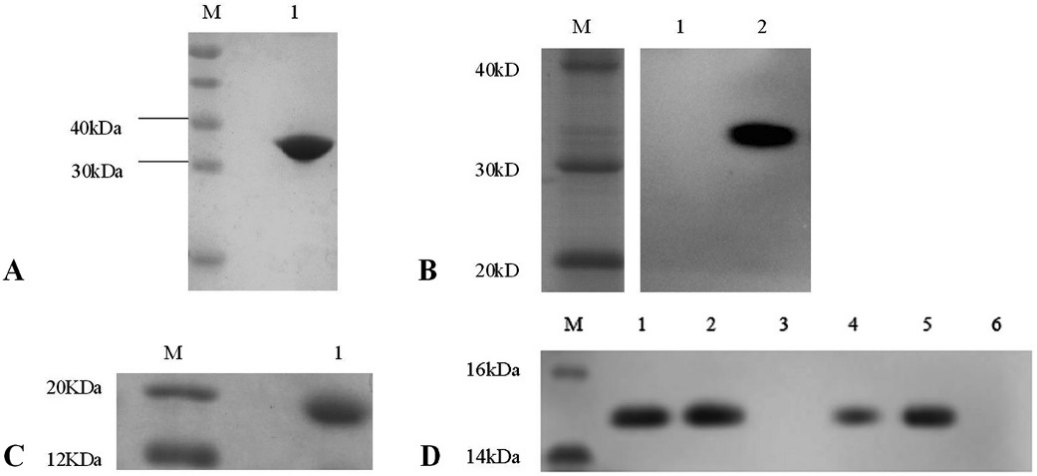
**Analysis of the His-tagged R-HCC and VHHs. (A)** SDS-PAGE analysis of recombinant His-HCC expressed in Rosetta (DE3), Lane M: the molecular weight markers; Lane1: purified His-tagged R-HCC. **(B)** Western blot identification of purified His-tagged HCC using mouse anti-His IgG, Lane 1: Control; Lane2: purified His-tagged R-HCC. **(C)** SDS-PAGE analysis of His-tagged R-HCC digested by thrombin and purified by Ni-NTA, Lane M: the molecular weight markers; Lane1: purified R-HCC. **(D)** Western blot analysis of purified VHH using mouse anti-His IgG, Lane M: the molecular weight markers; Lane1: purified VHH 3-2; Lane2: purified VHH 3-24; Lane 3: purified VHH 3**-**30; Lane 4: purified VHH 3**-**33; Lane 5: purified VHH 4**-**5; Lane 6: purified VHH 4**-**8.

The gene encoding HCC construct prokaryotic expression vector Pet-32a-HCC was cloned and expressed in prokaryotic system. The Figure 1A,B demonstrated the protein expression in insoluble inclusion bodies about 80%. R-HCC was obtained at a concentration of 2.377 mg/ml and the molecular weight of 17KD, of which the purity was more than 95% (Figure 1C) for McAbs and VHHs. Table 1 shows the titer of sera from mice, of which two mice were more suitable as a mouse hybridoma fusion after four injections of R-HCC.

**Table 1:** **Detection of mouse serum antibody titers by ELISA kit**

Number of mice	Titer
#1	128,000
#2	256,000
#3	128,000
#4	256,000

Of hybridoma McAbs, McAbs 5 F2, 4E4, or 1E11 had high affinity with the coefficients at 2.258^-9^, 2.975^-8^, or 3.622^-9^, respectively. The antibody heavy chain is γ chain subclasses (IgG1), while light chain subclasses are κ chains (Table 2). The steric orthe same decision epitopes of HCC was identified in those McAbs.

**Table 2:** **Characteristics of the anti-HCC McAbs**

The cell culture supernatant of HCC McAbs		
Number of clones	Titer (OD450 ± SD)	Antibody subtypes
5 F2	2.513 ± 0.179	IgG1,κ
4E4	2.634 ± 0.087	IgG1,κ
1E11	2.426 ± 0.081	IgG1,κ

1 × 10^6^ of each hybridoma, in 10 ml medium for 2 days, the supernatant was detected directly by ELISA. Data shown in the Table are the mean value of three independent experiments.

**Figure 2:**
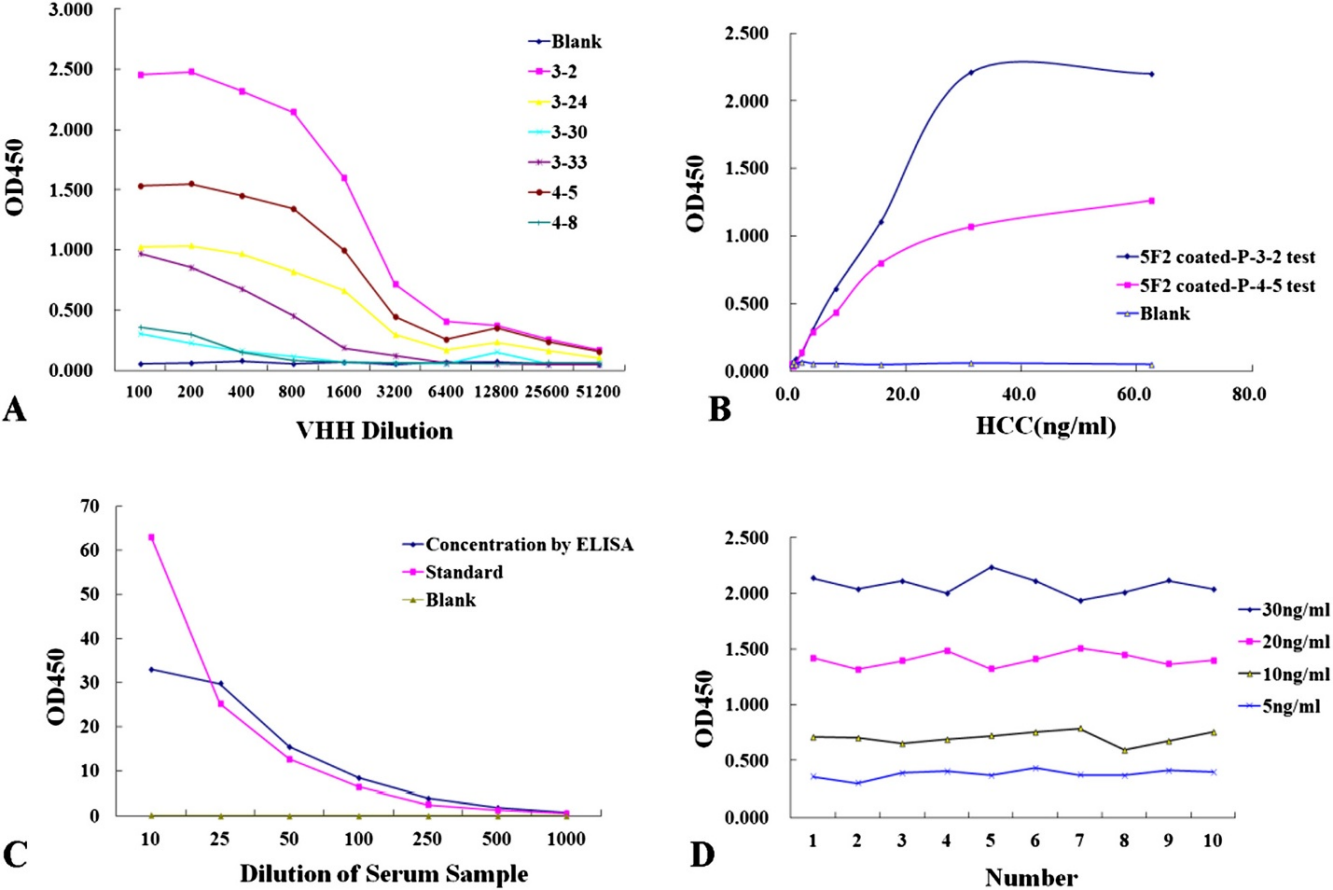
**Determination of VHHs’ Affinity, the establishment of DAS-ELISA and determination of assay properties. (A)** The affinity of VHHs was determined by ELISA, VHHs were serially diluted into PBS (0.1 M, pH7.4) and PBS was used as the blank control. Each point represents the mean value of triplicate determinations. **(B)** Detection of HCC by paired test, the best concentration of coated 5 F2 was 5 ug/ml. Each point represents the mean value of triplicate determinations. **(C)** Detection of serum HCC by established DAS-ELISA, comparison of HCC level in serum samples between ELISA value and the standard value. The HCC concentration of the serum samples by the ELISA kit is 0.71 μg/ml, the accuracy is high. **(D)** Precision was measured by ELISA kit, the figure showed that intra-variability CV were 4.1%, 4.5%, 7.94% and 10.1%, in line with the requirements of CV <15%.

Ten monoclonal colonies with the highest absorptiometry were picked from the third round and the fourth round respectively. Six different sequences numbered 3-2, 3-24, 3-30, 3-33, numbered 4-5, 4-8, were obtained, respectively, as shown in Table 3. The VHH genes were expressed in the prokaryotic system and purified. VHH 3-2, 3-24, 3-33, 4-5 could recognize the N-HCC specifically, with the highest affinity for 3-2 (Figure 2A), while VHH 3-30, 4-8 did not (Figure 1D).

**Table 3:** **Selection of VHH antibodies against HCC**

Number of clone	FR1	CDR1	FR2	CDR2
**3-2**	MADVQLQASGGGLVQAGGSLRLSCAAS	GSIVSIND	MGWYRQAPGKQRDLVAL	ITRGGNT
**3-24**	MADVQLQASGGGLVQPGGSLRLSCAVS	GTNFRLND	MAWYRQPPEKRRELVAL	ITGGGNT
**3-30**	MADVQLQASGGGLVQAGGSLRLSCAAS	GSIASIHD	MGWYRQTPGKQRDLVAL	ITRGGNT
**3-33**	MAEVQLQASGGGLVQPGGSLRLSCAAS	RMVIRTFSGAD	MGWYRQISRNQRELVAL	ITSGGNT
**4-5**	MAEVQLQASGGGLVQPGGSLRLSCAA	RMVFSTFSGAD	MGWYRQISGNQRELVAL	ITSGGNT
**4-8**	MAEVQLQASGGGLVQPGGSLRLSCAAS	GSIFSIND	MGWYRQAPGKQRELVAF	ITRGGNTH
**Number of clone**	**FR3**		**CDR3**	**FR4**
**3-2**	NYADSVKGRFTISRDNAKSTVYLQMNNLKPEDTAVYYC		ATLTRPAYW	GQGTLVTVSSGR
**3-24**	SYADSVKDRFTISRDNIQRTLYLQMNSLKPEDTAVYYC		TTQRSGRQYW	GKGTHVTVSSGR
**3-30**	NYADSVKGRFTISRDNAKSTVYLQMNSLKPEDTAVYYC		ATLTRPAYW	GQGTLVTVSSGR
**3-33**	NYTDSVKGRFTISRDNAKGTLYLQMSNLKPEDTAHYYC		AKISFTGPHRW	GQGTQVTVSSGR
**4-5**	NYTDSVKGRFTISRDNAKGTLYLQMSSLKPEDTAHYYC		AKISRTTPHYW	GQGTQVTVSSGR
**4-8**	HYADSAKGRFTISRDNAKNTLYLQMNSLKPEDTAVYYC		NTVNTRTRSW	GQGTQVTVSSGR

Amino acid sequences of the diverse binders selected after panning. FR (framework region) and CDR (complementarity determining region) were deduced according to Ana Monegal (**Ana Monegal, 2009**). Each VHH contains 4 FR and 3 CDR.

The paired results by sandwich ELISA between McAbs 5 F2, 4E4, or 1E11 were negative. The paired results between McAbs and VHHs (phages) demonstrated that McAb 5 F2 and VHH 3-2, 4-5 (VHH phages P-3-2 and P-4-5) were paired to detect HCC, and showed optimal effects (Figure 2B). The optimal coating concentration of 5 F2 and the optimal detection dilution of P-3-2 were 5 μg/ml and 1000 times. DAS-ELISA for HCC was established with the self-made McAbs and VHHs. The prepared McAbs 5 F2 and VHH phages P-3-2 or P-4-5 were applied for the development of the measurement. The optimal effect was achieved with the minimum detection of 0.5 ng/ml and the linear range of 0.5 ~ 30 ng/ml. The accuracy measured by adding HCC to the urine was listed in Table 4, and the precision measured by adding HCC to serum was shown in Figure 2C,D.

**Table 4:** **Recovery of HCC in spiked urine samples by ELISA kit**

Standard(ng/ml)	OD450	Added HCC (ng/ml)	OD450	Detected HCC (ng/ml)	Recovery
0	0.052	0	0.097	1.36	-
1	0.070	1	0.171	2.40	105.60%
2	0.137	2	0.249	3.50	107.20%
5	0.376	5	0.434	6.10	95.10%
10	0.690	10	0.840	11.80	104.40%
25	1.812	25	1.937	27.20	103.40%
30	2.201	30	2.113	29.68	94.40%

The accuracy of HCC detection kit is 94.8%^1^ from the data of the table.

^1^Accuracy = 1-{(105.6%-1) + (107.2%-1) + (1–95.1%) + (104.4%-1) + (103.4%-1) + (1–94.4%)}/6.

^2^ = **C**(control), “**C**” means concentration.

^3^Recovery = {**C**(Detected HCC) -**C**(control)}/**C**(Standard).

## Discussion

Cystain C, a 120 amino acid peptide chain with approximate 13KD, belongs to the family of papain-like cysteine proteases and has the biological role in the extracellular inhibition of Cathepsins. Cystain C is constantly and largely produced filtered from the glomerular membrane, and then completely reabsorbed from the proximal tubular cells. It has been considered as a biomarker candidate of renal function, although it is difficult to take the paired antibodies by hybridoma technology [40]. Diagnostic kits of cystatin C commercially available are mainly based on the traditional monoclonal antibody to hardly meet requirements of the clinical application. The present study developed a new way to detect human serum cystatin C with high sensitivity and specificity through the combination of hybridoma technology and phage display technology.

With the advantage of phage display it is easier than the traditional methods to get the paired antibody for detection of HCC and it provides a new strategy for the detection of HCC and other small molecules. Using R-HCC as a screening antigen and N-HCC as the detection of antigens, we successfully obtained McAbs and VHHs against N-HCC with high affinity. The optimal effect can be achieved with the detectable minimum of 0.5 ng/ml and the detecting linear range of 0.5 ~ 30 ng/ml, through continuous optimization of conditions. The developed ELISA kit can be used to quantitative detection of HCC in human urine and human serum with the CV precision <15%. Such pairing can be used for other HCC quantitative measurements, such as colloidal gold strip or latex particle turbidimetric immunoassay method.

HCC can be stable at room temperature for 2 days, 0 ~ 20°C for 7 days, or -80°C for 6 months, without the influences from repeated freezing and thawing. The present study confirmed the reliability and authenticity of the HCC determination, so further improvements of HCC measurements can lead to potential social and economic significance. The combination of the hybridoma technique and phage display technology for screening of paired antibodies provides a new solution, with great biological and clinical significance. The cystain C as a biochemical marker or predictor of GFR offers the greater sensitivity in detecting an abnormal GFR [41, 42]. The present study provides the new measurement of GFR to monitor the occurrence and progress of renal diseases. The further works on the application of the clinical measurement of renal disease and other diseases are needed [43–45].

## Conclusions

The present study developed and validated a new measurement of HCC as a biomarker for clinical measurement of renal diseases, and established DAS-ELISA for HCC with the self-made McAbs and VHHs by applying the hybridoma technology and phage VHH display technology with high sensitivity.

## Abbreviations

CV: Coefficient of variation
DAS-ELISA: Double-antibody-sandwich enzyme-linked immunosorbent assay
GFR: Glomerular filtration rate
HAT: Hypoxanthine-aminopterin-thymidine supplemented medium
HT: Hypoxanthine-thymidine supplemented medium
IPTG: Isopropyl thio-β-D-galactose glycoside
Kd: Dissociation constant
LB: Luria-Bertani
McAb: Monoclonal antibody
MW: Molecular weight
N-HCC: Natural human cystatin C
OD: Optical density
PEG: Polyethylene glycol
PMSF: Phenylmethanesulfonyl fluoride
R-HCC: Recombinant Human cystatin C
SDS-PAGE: sodium dodecyl sulfate polyacrylamide gel electrophoresis
VHH: Variable domain of heavy chain of heavy-chain antibody.
